# Etiology and therapy of pharyngeal perforations

**DOI:** 10.1007/s00405-024-09115-9

**Published:** 2024-12-04

**Authors:** Givi Magradze, Andreas Knopf, Christoph Becker, Manuel Christoph Ketterer

**Affiliations:** 1 Department of Otorhinolaryngology, Medical Center– University of Freiburg, Faculty of Medicine, University of Freiburg, Freiburg, Germany; 2 Department of Otorhinolaryngology– Head and Neck Surgery, University Medical Centre Freiburg, Killianstrasse 5, 79106 Freiburg, Germany

**Keywords:** Hypopharynx, Iatrogenic perforation, Perforation management, Pharynx

## Abstract

**Objective:**

The primary aim of this study is to evaluate the impact of diagnostic procedures and treatment interventions performed at our medical institution on the final outcomes and survival rates of patients with iatrogenic and traumatic pharyngeal perforation (PP).

**Materials and methods:**

We reviewed the medical records of 36 patients with iatrogenic and trauma-induced PP who were treated at the Quaternary Medical Center of Otorhinolaryngology between 2010 and 2020. Comorbidities were classified according to the Age-adjusted Charlson Comorbidity Index (ACCI) scoring system, and postoperative complications were classified according to the Clavien and Dindo scoring system.

**Results:**

Of the 36 patients, 15 (41.7%) were male and 21 (58.3%) were female. The median age was 73 years, and PP was typically diagnosed within one day. Notably, the perforation site was identified in the hypopharynx in 29 (80.5%) patients. The median ACCI score was 4, with the most frequent ACCI score observed being 5. During the treatment course, 17 patients (47.2%) experienced complications, with 9 of these patients experiencing grade IV complications according to the Clavien and Dindo classification.

**Conclusion:**

Our study showed that patients with hypopharyngeal perforations have an almost 42-fold higher risk of mortality during hospitalization compared to those with epipharyngeal or oropharyngeal perforations, though results are limited by the small sample size and the variable dates. Additionally, neurosurgery of the cervical spine, transesophageal echocardiography, and diverticular surgery emerged as procedures carrying the highest risk for pharyngeal perforations. Within our patient cohort, 4 patients (11.11%, all female) died during the treatment course.

## Introduction

Pharyngeal perforation (PP) is a rare but life-threatening condition with controversy surrounding its treatment and diagnosis, necessitating early detection and personalized therapy [1, 2]. Yet, there is a scarcity of literature specifically addressing PP.

PP most commonly occurs during routine medical procedures such as pharyngoscopy, laryngoscopy, rigid esophagoscopy, transesophageal echocardiography, esophagogastroduodenoscopy, and transoral or nasotracheal intubation [3]. However, PP can lead to significant, potentially life-threatening complications such as mediastinitis, neck abscess, and sepsis [4–6].

The management of PP is a complex process involving numerous steps. The gold standard for diagnosing PP is imaging by contrast esophagography using a water-soluble contrast agent [7]. Depending on the location, size of the injury, and cause, a decision is made for conservative or surgical therapy [8].

Antibiotic therapy and nasogastric tube placement are important components of PP management [2, 9]. The aim of this study is to evaluate risk factors and improve the diagnosis and treatment of patients with PP.

## Materials and methods

The study was performed in accordance with the guidelines of the Helsinki Declaration of 1975, as revised in 2013 and the study protocol was approved prior to data collection by the local institutional review board (Ethics Committee of the Albert-Ludwigs-University of Freiburg number: 258/20). The study was also registered in the German Clinical Trials Register (DRKS00025435).

Incident cases of PP between 2010 and 2020 treated at the University Medical Centre Freiburg, Department of Otorhinolaryngology were identified. Subsequently, patient characteristics such as age and gender as well as comorbidities were systematically recorded by retrospective review of patient records. In addition to this demographic information, details of the characteristics of the perforation, including its location and treatment strategies used, were meticulously extracted. Comprehensive follow-up data were also collected, encompassing various aspects of the patient’s health history. These included, for example, laboratory information at perforation and during follow-up, length of stay in the intensive care unit, blood transfusions, duration of antibiotic therapy, number of surgical procedures performed, interval between diagnosis and surgery, duration of nasogastric tube placement, and total length of hospital stay.

Comorbidities were classified according to the Age-Adjusted Charlson Comorbidity index (ACCI) [10]. ACCI is a scoring system that estimates approximate mortality for patients with 19 underlying diseases. ACCI is based on information from medical records [10]. The index is widely used, and its effectiveness in classifying comorbidities has been validated for several disease subgroups [11–13].

Anemia was defined as hemoglobin < 13.5 g/dL or hematocrit (HCT) < 41.0% in men, and hemoglobin < 12.0 g/dL or HCT < 36.0% in women as discussed by Williams published 2006 [37]. Anemia is associated with poorer surgical results and outcomes in a variety of diseases such as hypopharyngeal, laryngeal and sinonasal carcinoma [12, 14–16]. 

Complications were classified using the Clavien-Dindo classification of surgical complications. First established in 1992, Clavien et al. proposed a review version of this classification system in 2004. To revalidate this classification, Clavien et al. reviewed 650 cases of elective cholecystectomy and demonstrated its correctness [17]. In the modified classification of 2004 there are 5 grading systems, in the old version of 1992 there were only 4. Validation of the modified classification was performed on a cohort of 6336 patients [18]. The Clavien-Dindo classification serves as a useful, highly objective tool for classifying complications after surgical procedures [19].

The statistical analysis was conducted using IBM SPSS Version 29 Statistics. Descriptive statistics, including minimum, maximum, and median values, are presented both in tables and within the text for comprehensive understanding. Cox Regression analysis, employing forward selection in a multivariate approach, was performed to assess the impact of diagnostic and treatment interventions on patient outcomes and survival. The variables considered in the multivariate analysis included the perforation site, hemoglobin levels, Age-adjusted Charlson Comorbidity Index (ACCI), and leukocyte counts. T-values, confidence intervals, and hazard ratios were computed as part of the Cox Regression analysis. Significance levels were set at *p* < 0.05, ensuring robust statistical evaluation. This approach allowed for a nuanced exploration of the interplay between various factors and their influence on both the outcome and survival of patients with pharyngeal perforation.

## Results

### Patients and diagnosis

**Fig. 1:**
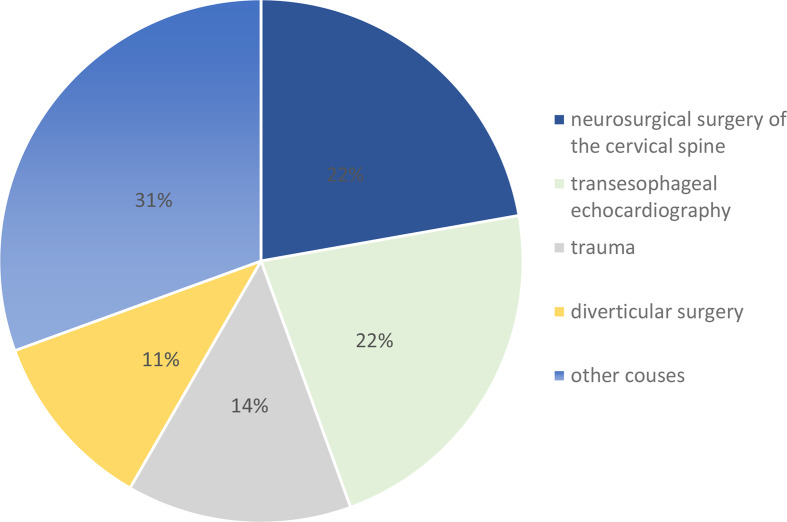
Graphic representation of the percentage breakdown of the causes of perforations

Thirty-six patients were included in the study, with a higher number of female patients (*n* = 21, 58.3%). The median age at PP diagnosis was 73 years (range 19–91 years, Table 1). Twenty-three patients (63.8%) were older than 65 years old. The median ACCI score was 4, and the most frequent ACCI score was 5. The majority of perforations were caused by neurosurgical surgery of the cervical spine (*n* = 8, 22%), transesophageal echocardiography (*n* = 8, 22%), trauma (*n* = 5, 13.9%), or diverticular surgery (*n* = 4, 11.1%, Fig. 1). Hypopharyngeal perforation was found in 29 (80.5%) patients. The interval between perforation and diagnosis varied from 0 to 6 days. 63.8% of the diagnoses (23 patients) were made on the same day. Twenty-nine patients (80.5%) initially underwent a computed tomography scan for diagnosis. In 25 patients (69.4%), computed tomography was also performed for follow-up. Twenty-three patients (63.8%) received control imaging by contrast esophagography with water-soluble contrast. Additionally, a detailed analysis was performed to explore the percentage and median differences in patient age, age-adjusted Charlson Comorbidity Index (ACCI), laboratory findings, invasive ventilation, ICU stay, and blood transfusion between patients with epi-/oropharyngeal and hypopharyngeal perforations (refer to Table 2), revealing notable distinctions without statistical significance.

**Table 1:** This table represents minimum, maximum, and median data of patient age, age-adjusted Charlson Comorbidity Index (ACCI) score, labor findings, conservative therapy duration, number of surgical procedures, interval between diagnosis and surgery, and hospitality duration

	*N*	Minimum	Maximum	Median
Age	36	19	91	73
ACCI	36	0	8	4
Duration of antibiotic therapy	32	1	59	12.5
Leucocytes on the day of the perforation	36	2.57	36.77	11.88
Leucocytes during the course	35	2.08	15.46	7.32
Hemoglobin at perforation	36	6.60	16.40	11.9
C-reactive protein on the day of the perforation	23	< 3	495.00	63.5
C-reactive protein during the course	29	< 3	108.00	34
Number of surgical procedures	36	1	10	2.7
Interval between diagnosis and surgery	36	0	15	0.5
Duration of a nasogastric tube	34	0	49	10
Duration of hospitality	36	2	78	17.5

**Table 2:** This table presents the percent and median data difference of patient age, age-adjusted Charlson Comorbidity Index (ACCI), laboratory findings, invasive ventilation, ICU therapy, and blood transfusion between patients with perforation in the epi-/oropharynx and hypopharynx

	Epi-/Oropharynx	Hypopharynx	*p*
Number of patients	7 (♂/♀)	29 (♂/♀)	
Age (median)	55	74	0.047
ACCI (median)	3	5	0.144
Leucocytes at event of PP (median)	11.9	11.8	0.475
Hemoglobin at perforation (median)	11.4	12.1	0.258
Invasive ventilation (n/%)	4 (57%)	13 (45%)	0.286
Intensive care unit (n/%)	7 (100%)	21 (72%)	0.001
Blood transfusion (n/%)	0	9 (31%)	0.001

## Treatment

Thirty-five patients (97%) were treated with antibiotics. Twenty-two patients (61%) were treated with cephalosporins in combination with metronidazole. The duration of antibiotic therapy varied from 1 to 59 days, with a median duration of 12.5 days. Twenty-eight patients (77%) were treated in the intensive care unit. Nine patients (25%) received blood transfusions. Thirty-six patients (100%) underwent surgical treatment. In the analysis of the initial procedures performed, the frequently performed procedure was transcervical mucosal suture, which was carried out in 19 (52.78%) patients. This was followed by transoral suture alone, performed in 6 (16.67%) patients. Other procedures included transcervical mucosal sutures with flap closure techniques, such as pectoralis and free flap procedures in 2 (5.56%) patients, and thoracoscopy/thoracotomy/mediastinotomy in 2 (5.56%) patients. Additionally, panendoscopy with neck exploration and mediastinotomy was conducted in 1 (2.78%) patient, while removal of cervical spine plating and stent insertion were each performed in 1 (2.78%) patient. Finally, panendoscopy or pharyngoscopy was conducted in 4 (11.11%) patients. Surgical intervention as a first and repeat procedure was performed in 4 (11%) patients using transcervical mucosal sutures with flap closure techniques such as pectoralis and free flap procedures. The number of surgical procedures performed varied between 1 and 10, depending on complications after the first surgery. The average number of surgical procedures was 2.7. Twenty-one patients (58.3%) suffered from anemia. Thirty-four patients (94.4%) were fed via a gastric tube, while in the remaining two patients (5.6%), a percutaneous endoscopic gastrostomy had already been performed before the perforation. The average duration of feeding via a nasogastric tube was 12.24 days.

## Complications

**Fig. 2:**
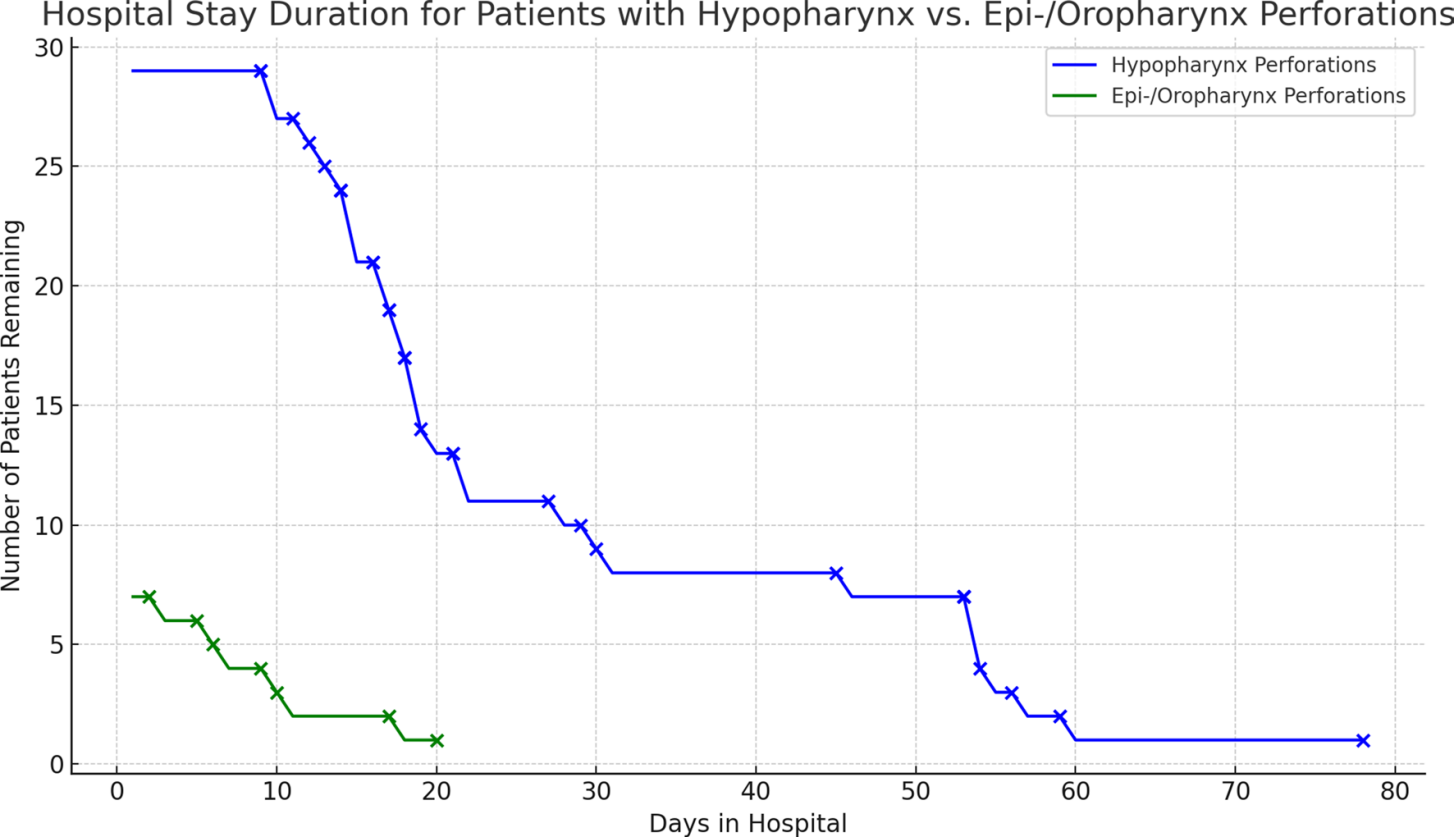
This line chart illustrates the hospital stay duration for patients with hypopharyngeal perforations (blue line) compared to those with epi-/oropharyngeal perforations (green line). The continuous lines represent the number of patients remaining in the hospital over time. Points along the lines indicate the exact days when patients were either discharged or deceased

Seventeen patients (47.2%) developed complications during the course of treatment. Nine out of 17 patients with complications suffered from a complication grade IV according to Clavien and Dindo. Two out of 17 patients developed a grade II complication, as did another two patients out of 17 who experienced a grade III complication according to Clavien and Dindo. Four patients (11.1%) died during their treatment course (please find Table 3). All four patients who died were women. Table 3 shows a detailed distribution of the deceased and surviving patients. Nevertheless, the defect size is only known in 1 deceased and 10 surviving patients. In the group of patients who survived, C-reactive protein (CRP) was known 48 h after perforation in only 10 patients. In contrast, in the group of patients who died, CRP was not evaluated 48 h after perforation. The median age at death was 76 years (range 62–91 years). The median ACCI score of the deceased patients was 6.5. The ACCI score in two of the deceased patients was 8. Septic shock developed in three of the deceased patients, partly with multiple organ failure. The median hospital stay of all patients was 17.5 days (range 2 to 78 days, Fig. 2).

**Table 3:** The table shows the median of the following parameters: defect size in cm, difference between diagnosis and operation date in days, anaemia (haemoglobin value), ACCI (Age-adjust Charlson Comorbidity Index), as well as the CRP value on the day of perforation and 48 h after perforation. In addition, the median leucocyte count on the day of perforation and 48 h after perforation is shown

	Deceased patients	Surviving patients
Location of perforation (hypopharynx) in %	3 (75%)	26 (81.2%)
Location of perforation (Epi-/Oropharynx) in %	1 (25%)	6 (18.8%)
Size of defect in cm (median)	5	2.5
Time to diagnosis in days (median)	1	0
Anemia on the day of the perforation (HB) (median)	10.6	12.1
ACCI (median)	6.5	4
CRP on the day of the perforation (median)	103	45.4
CRP in 48 h	x	53.2
Leucocytes on the day of the perforation (median)	10.94	11.85
Leucocytes in 48 h (median)	9.4	8.84

The hazard ratio analysis revealed that the risk of mortality during hospital stay for patients with hypopharyngeal perforations is nearly 42 times higher than for those with epipharyngeal or oropharyngeal perforations. However, it is important to interpret these findings with caution due to limitations associated with the small sample size and variable dates, which may impact the robustness and generalizability of the results. Correlation analysis revealed a weak positive relationship between the time elapsed between perforation and diagnosis and mortality. Nevertheless, we could not calculate a significant correlation. Furthermore, we did not find a significant relationship between the cause of perforation (open vs. trauma vs. endoscopic) and mortality.

## Discussion

PP is a challenging clinical entity that requires careful consideration regarding diagnosis and treatment. The rarity of PP and the diversity of its causative factors underscore the need for dedicated research and differentiated approaches to improve patient outcomes [1, 2].

Our study, focused on iatrogenic and traumatic PP, delves into the complexities surrounding diagnostic and treatment interventions. The comprehensive review of medical records from 36 patients treated at the Quaternary Medical Center of Otorhinolaryngology between 2010 and 2020 forms the basis of our insights.

Our findings suggest an association between the location of pharyngeal perforation and the risk of mortality during hospitalization. Specifically, patients with hypopharyngeal perforations have an almost 42-fold higher risk of mortality compared to those with epipharyngeal or oropharyngeal perforations. This emphasizes the need for tailored interventions, vigilant monitoring, and potentially different treatment modalities based on the perforation site. However, these results should be interpreted with caution due to the limited patient sample.

PP may be suspected from the history and findings on physical examination, but the diagnosis depends on imaging and endoscopic examination [4, 7]. If a perforation is identified endoscopically, it may also be closed simultaneously with an endoscopic closure technique, depending on certain criteria such as the location, size and margins of the defect. Endoscopic closure techniques include Through-the-Scope-Clips, Over-the-Scope-Clips, esophageal stents and endoscopic suturing techniques [20].

The gold standard for the diagnosis of PP is imaging by contrast esophagography with a water-soluble contrast agent. Barium provides better contrast images, but may cause a more severe inflammatory reaction, therefore water-soluble contrast media with minimal mediastinal irritation is preferred [7, 21–23].

Computed tomography with oral or intravenous contrast has a high sensitivity. CT with contrast media should be considered as the first imaging test for PP [24, 25].

Our study shows that computed tomography was initially performed for diagnosis in 29 patients (80.5%), whereas contrast esophagography with a water-soluble contrast agent was not used as initial diagnosis. Contrast esophagography with a water-soluble contrast agent was only performed as control imaging. Computed tomography was used not only for initial diagnosis but also for follow-up in 25 patients (69.4%).

A quick and correct diagnosis is very important, a delayed diagnosis leads to a delayed therapy and a therapy delayed more than 24 h can lead to a higher morbidity [26–29]. A delay in treatment of 12–24 h allows sufficient leakage of saliva and bacteria surrounding loose areolar tissue to produce a purulent infection and an intensifying inflammatory response [30]. This inflammation in the fascial spaces of the head and neck may allow rapid downward spread of infection into the mediastinum under gravity and negative intrathoracic pressure [31].

In our study, the interval between perforation or event that caused perforation and diagnosis was between 0 and 6 days. The interval was between 1 and 6 days in 13 patients (36%), in other 23 patients (63.88%) perforation was identified on the same day.

Due to a lack of sufficient studies, the treatment method for perforations is still controversial today. After an adequate diagnosis, it is important to start therapy quickly. Whether conservative therapy or surgical therapy, the nasogastric feeding tube is one of the most important procedures for therapy of PP, which can also be inserted during panendoscopy [9]. The most commonly used antibiotics in the treatment of PP are cefuroxime and metronidazole as well as imipenem and meropenem [2]. Cefuroxime is effective against most gram-negative and gram-positive aerobes and metronidazole is highly effective against anaerobes [32, 33].

According to our data, all 36 patients (100%) were treated surgically. The most frequently performed procedure was transcervical mucosal suture (*n* = 19/52.78%). Surgical intervention as a first and repeat procedure together was performed in 4 (11%) patients using transcervical mucosal sutures with flap closure techniques such as pectoralis and free flap procedures. Despite initial adequate surgical and antibiotic therapy as well as feeding via nasogastric tube, surgical interventions should have been performed again in 15 patients (41.66%). The number of surgical interventions performed varied from 1 to 10, depending on the complications after the initial surgery. Cephalosporins in combination with metronidazole were used in 22 patients (61%). A total of 35 patients (97%) were treated with antibiotics.

Depending on localization perforations smaller than 2 cm can be treated conservatively with observation, gastric tube and intravenous antibiotics; however, if lesions are larger than 2 cm, surgical therapy, if necessary, neck exploration with drainage is recommended [8].

After approximately 5–21 days after insertion of the gastric tube in the case of surgical or conservative therapy before removal of a gastric tube, a contrast esophagography with a water-soluble contrast medium is recommended to exclude extravasation [34].

Our retrospective study showed that mortality was rather low compared to other studies. However, the management of PP is still controversial until now. Collaborative efforts, prospective studies, and multicenter investigations are essential to develop more accurate concepts and recommendations for the diagnosis and treatment of pharyngeal perforations.

## Conclusion

PP in adults is a highly morbid disease with high mortality rate of approximately 20%. Key prognostic factors include the timing of presentation, the location and etiology of the perforation, and delays in diagnosis and treatment. Hypopharyngeal perforations, in particular, demonstrate high mortality, necessitating individualized treatment approaches. Mortality rates range from 10 to 25% when treatment starts within 24 h but can rise to 40–60% if delayed beyond 48 h [26, 35, 36]. Our study identified cervical spine neurosurgery, transesophageal echocardiography, and diverticular surgery as the most common causes of PP, with a mortality rate of 11.11% among the patients studied.

## Data Availability

To protect the privacy of study participants and for ethical reasons, the data cannot be shared openly but can be requested from the corresponding author.

## References

[1] Chen S, Shapira-Galitz Y, Garber D, Amin MR (2020) Management of iatrogenic cervical esophageal perforations: a narrative review. JAMA Otolaryngol - Head Neck Surg 146(5):488–494. 10.1001/jamaoto.2020.0088 32191285 10.1001/jamaoto.2020.0088

[2] Hinojar AG, Díaz Díaz MÁ, Pun YW, Hinojar AA (2003) Management of hypopharyngeal and cervical oesophageal perforations. Auris Nasus Larynx 30(2):175–182. 10.1016/S0385-8146(03)00042-7 12753990 10.1016/s0385-8146(03)00042-7

[3] Greer D, Marshall KE, Bevans S, Standlee A, McAdams P, Harsha W (2017) Review of videolaryngoscopy pharyngeal wall injuries. Laryngoscope 127(2):349–353. 10.1002/lary.26134 27345583 10.1002/lary.26134

[4] Onishi T, Onishi Y, Tachibana K et al (2014) Perforation of the hypopharynx after transesophageal echocardiography. J Echocardiogr 12(2):71–74. 10.1007/s12574-014-0213-5 27279053 10.1007/s12574-014-0213-5

[5] Eichborn KWG, Bley TA, Ridder GJ (2003) Unerkannte hypopharynx-perforation mit tiefem halsabszess und mediastinitis infolge transösophagealer echokardiographie. HNO 51(11):903–907. 10.1007/s00106-003-0809-y 14605709 10.1007/s00106-003-0809-y

[6] Kang MS, Kim KH, Park JY et al (2017) Management of esophageal and pharyngeal perforation as complications of anterior cervical spine surgery. World Neurosurg 102:275–283. 10.1016/j.wneu.2017.02.130 28286279 10.1016/j.wneu.2017.02.130

[7] Brinster CJ, Singhal S, Lee L, Marshall MB, Kaiser LR, Kucharczuk JC (2004) Evolving options in the management of esophageal perforation. Ann Thorac Surg 77(4):1475–1483. 10.1016/j.athoracsur.2003.08.037 15063302 10.1016/j.athoracsur.2003.08.037

[8] Kesser BW, Chance E, Kleiner D, Young JS (2009) Contemporary management of penetrating neck trauma. Am Surg 75(1):1–10. 10.1177/000313480907500101 19213388

[9] Mao JC, Kayali FM, Dworkin JP, Stachler RJ, Mathog RH (2009) Conservative management of iatrogenic esophageal perforation in head and neck cancer patients with esophageal stricture. Otolaryngol - Head Neck Surg 140(4):505–511. 10.1016/j.otohns.2008.12.052 19328338 10.1016/j.otohns.2008.12.052

[10] Charlson M, Szatrowski TP, Peterson J, Gold J (1994) Validation of a combined comorbidity index. J Clin Epidemiol 47(11):1245–1251. 10.1016/0895-4356(94)90129-5 7722560 10.1016/0895-4356(94)90129-5

[11] Yang CC, Fong Y, Lin LC et al (2018) The age-adjusted Charlson comorbidity index is a better predictor of survival in operated lung cancer patients than the Charlson and Elixhauser comorbidity indices. Eur J Cardio-thoracic Surg 53(1):235–240. 10.1093/ejcts/ezx215 10.1093/ejcts/ezx215 29106506

[12] Becker C, Dahlem KKK, Lange K, Ketterer MC, Pfeiffer J (2018) Predictive value of comorbidity and anemia on outcome in patients with sinonasal carcinoma. J Cranio-Maxillofacial Surg 46(8):1373–1378. 10.1016/j.jcms.2018.05.009 10.1016/j.jcms.2018.05.009 29807754

[13] Sorror ML, Maris MB, Storb R et al Hematopoietic cell transplantation (HCT)-specific comorbidity index: a new tool for risk assessment before allogeneic HCT. Published online 2005. 10.1182/blood-2005-05-2004 10.1182/blood-2005-05-2004 PMC1895304 15994282

[14] Kansagra AJ, Stefan MS (2016) Preoperative Anemia. Evaluation and treatment. Anesthesiol Clin 34(1):127–141. 10.1016/j.anclin.2015.10.011 26927743 10.1016/j.anclin.2015.10.011

[15] Clevenger B, Mallett SV, Klein AA, Richards T (2015) Patient blood management to reduce surgical risk. Br J Surg 102(11):1325–1337. 10.1002/bjs.9898 26313653 10.1002/bjs.9898

[16] Ketterer MC, Lemus Moraga LA, Beitinger U, Pfeiffer J, Knopf A, Becker C (2020) Surgical nodal management in hypopharyngeal and laryngeal cancer. Eur Arch Oto-Rhino-Laryngology 277(5):1481–1489. 10.1007/s00405-020-05838-7 10.1007/s00405-020-05838-7 PMC7160213 32048029

[17] Clavien PA, Sanabria JR, Strasberg SM (1992) Proposed classification of complications of surgery with examples of utility in cholecystectomy. Surgery 111(5):518–526. https://pubmed.ncbi.nlm.nih.gov/1598671/ Accessed October 11, 2023 1598671

[18] Dindo D, Demartines N, Clavien P-A, Clavien P-A (2004) Classification of Surgical complications a new proposal with evaluation in a cohort of 6336 patients and results of a survey. Ann Surg • 240(2). 10.1097/01.sla.0000133083.54934.ae 10.1097/01.sla.0000133083.54934.ae PMC1360123 15273542

[19] Miyamoto S, Nakao J, Higashino T, Yoshimoto S, Hayashi R, Sakuraba M (2019) Clavien-Dindo classification for grading complications after total pharyngolaryngectomy and free jejunum transfer. PLoS ONE 14(9). 10.1371/journal.pone.0222570 10.1371/journal.pone.0222570 PMC6742376 31513680

[20] Gurwara S, Clayton S (2019) Esophageal perforations: an Endoscopic Approach to Management. Curr Gastroenterol Rep 21(11):57. 10.1007/s11894-019-0730-5 31749030 10.1007/s11894-019-0730-5

[21] Keberle M, Wittenberg G, Trusen A, Hoppe F, Hahn D (2000) Detection of pharyngeal perforation: comparison of aqueous and barium-containing contrast agents. Am J Roentgenol 175(5):1435–1438. 10.2214/ajr.175.5.1751435 11044058 10.2214/ajr.175.5.1751435

[22] James AE, Montali RJ, Chaffee V, Strecker EP, Vessal K (1975) Barium or gastrografin: which contrast media for diagnosis of esophageal tears? *Gastroenterology*. 68(5). Accessed April 25, 2021. https://pubmed.ncbi.nlm.nih.gov/1126592/1126592

[23] Chen A, Tafti D, Tuma F, Barium, Swallow (2022) *StatPearls*. Published online September 7, Accessed November 3, 2022. PMID: 29630228

[24] Wei CJ, Levenson RB, Lee KS (2020) Diagnostic utility of CT and fluoroscopic esophagography for suspected esophageal perforation in the emergency department. Am J Roentgenol 215(3):631–638. 10.2214/AJR.19.22166 32515607 10.2214/AJR.19.22166

[25] Fadoo F, Ruiz DE, Dawn SK, Webb WR, Gotway MB (2004) Helical CT esophagography for the evaluation of suspected esophageal perforation or rupture. Am J Roentgenol 182(5):1177–1179. 10.2214/ajr.182.5.1821177 15100114 10.2214/ajr.182.5.1821177

[26] Jones WG, Ginsberg RJ (1992) Esophageal perforation: a continuing challenge. Ann Thorac Surg 53(3):534–543. 10.1016/0003-4975(92)90294-E 1489367 10.1016/0003-4975(92)90294-e

[27] Tracy LF, Piraquive J, Grillone GA (2020) Penetrating trauma of the Pharynx and Esophagus. Oper Tech Otolaryngol Neck Surg 31(4):332–338. 10.1016/j.otot.2020.10.011

[28] Amir AI, Dullemen HV, Plukker JTM (2004) Selective approach in the treatment of esophageal perforations. Scand J Gastroenterol 39(5):418–422. 10.1080/00365520410004316 15180177 10.1080/00365520410004316

[29] Asensio JA, Berne J, Demetriades D et al (1997) Penetrating esophageal injuries: time interval of safety for preoperative evaluation - how long is safe? J Trauma - Inj Infect Crit Care 43(2):319–324. 10.1097/00005373-199708000-00018 10.1097/00005373-199708000-00018 9291379

[30] Armstrong WB, Detar TR, Stanley RB (1994) Diagnosis and management of external penetrating cervical esophageal injuries. Ann Otol Rhinol Laryngol 103(11):863–871. 10.1177/000348949410301107 7979000 10.1177/000348949410301107

[31] Kocher GJ, Hoksch B, Caversaccio M, Wiegand J, Schmid RA, Schmid RA (2012) Diffuse descending necrotizing mediastinitis: surgical therapy and outcome in a single-centre series. Published Online. 10.1093/ejcts/ezs385 10.1093/ejcts/ezs385 22761501

[32] Neu HC, Fu KP (1978) Cefuroxime, a beta-lactamase-resistant cephalosporin with a broad spectrum of gram-positive and -negative activity. Antimicrob Agents Chemother 13(4):657–664. 10.1128/AAC.13.4.657 248268 10.1128/aac.13.4.657 PMC352306

[33] Freeman CD, Klutman NE, Lamp KC (1997) Metronidazole. A therapeutic review and update. Drugs 54(5):679–708. 10.2165/00003495-199754050-00003 9360057 10.2165/00003495-199754050-00003

[34] Madiba TE, Muckart DJJ (2003) Penetrating injuries to the cervical oesophagus: is routine exploration mandatory? Ann R Coll Surg Engl 85(3):162–166. 10.1308/003588403321661307 12831487 10.1308/003588403321661307 PMC1964361

[35] Kaman L, Iqbal J, Kundil B, Kochhar R (2010) Press Elmer Management of esophageal perforation in adults. Gastroenterol Res • 3(6):235–244. 10.4021/gr263w 10.4021/gr263w PMC5139851 27942303

[36] Gupta NM, Kaman L (2004) Personal management of 57 consecutive patients with esophageal perforation. Am J Surg 187(1):58–63. 10.1016/j.amjsurg.2002.11.004 14706587 10.1016/j.amjsurg.2002.11.004

[37] Williams’ Hematology 7th ed, Lichtman MA, Beutler E, Kipps TJ (eds)(2006) McGraw-Hill, New York

